# CD26 as a potential therapeutic target for lung adenocarcinoma

**DOI:** 10.3389/fonc.2025.1552587

**Published:** 2025-12-04

**Authors:** Wolfgang Jungraithmayr, Birte Ohm, Martina Haberecker, Alessandra Curioni-Fontecedro, Florian Janker, Ignacio Gil-Bazo, Alex Soltermann, Michaela Kirschner, Walter Weder, Isabelle Opitz, Jae-Hwi Jang

**Affiliations:** 1 Department of Thoracic Surgery, University Hospital Zurich, Zurich, Switzerland; 2 Department of Thoracic Surgery, Medical Center – University of Freiburg, Faculty of Medicine, University of Freiburg, Freiburg, Germany; 3 Division of Thoracic Surgery, Rostock University Medical Center, Rostock, Germany; 4 Department of Pathology and Molecular Pathology, University Hospital Zurich, Zurich, Switzerland; 5 Medical Oncology, Cantonal Hospital Fribourg, University of Fribourg, Fribourg, Switzerland; 6 Department of Oncology, Clinica Universidad de Navarra, Pamplona, Spain; 7 Fundación Instituto Valenciano de Oncología, Valencia, Spain; 8 Centro de Investigación Biomédica en Red Cáncer (CIBERONC), Madrid, Spain

**Keywords:** CD26, DPP4, lung cancer, EMT, adenocarcinoma

## Abstract

**Introduction:**

CD26/dipeptidyl peptidase 4 (CD26, DPP4) is a transmembrane exopeptidase that modulates tumorigenesis in different malignancies. We demonstrated before that CD26 inhibition decreases lung tumor growth in experimental models. Here, we analyzed the prognostic significance of CD26 expression and its correlation with epithelial-to-mesenchymal transition (EMT) markers in a large series of patients with non-small cell lung cancer (NSCLC).

**Patients and methods:**

NSCLC samples from operated patients were analyzed using immunohistochemistry (IHC) for the expression of CD26 and EMT markers. CD26 was scored semi-quantitatively employing tissue microarrays. Lung cancer cell lines [H460, Lewis lung carcinoma (LLC)] were tested for EMT markers, and a colony formation assay was used to test the effect of treatment with the CD26 inhibitor vildagliptin.

**Results:**

Tumor samples from 904 patients with NSCLC were analyzed. CD26 IHC expression was significantly higher in adenocarcinoma compared to squamous cell carcinoma (p < 0.0001). Patients with adenocarcinoma and CD26 expression had a better overall survival than patients without CD26 expression. The lack of CD26 expression was shown to be an independent risk factor for worse survival. CD26-expressing adenocarcinomas showed a higher expression of Vimentin and Elastin (p = 0.0027 and p < 0.0001, respectively), while E-cadherin expression was lower in this group of patients (p = 0.0021). *In vitro*, treatment with vildagliptin reduced the expression of Vimentin and the capacity for colony formation in H460 and LLC cell lines.

**Summary and conclusion:**

The correlation of CD26 expression in lung adenocarcinomas and better patient survival, the antiproliferative effect on tumor cells by CD26 inhibition, and an altered EMT status give rise to the hypothesis that CD26 inhibitors impact the biology and clinical course of lung adenocarcinomas.

## Introduction

Lung cancer is the leading cause of death among all malignancies, accounting for more than 1.8 million deaths per year worldwide (1). Approximately 57% of all lung cancer cases are diagnosed at stage IV, showing a 5-year survival rate below 8% (2). Although surgery, chemo-radiotherapy, targeted therapy, and immunotherapy improved the overall survival of lung cancer patients, the prognosis remains poor (3).

CD26/dipeptidylpeptidase 4 (CD26, DPP4) is a multifunctional glycoprotein involved in glucose metabolism, immunomodulation, and tumorigenesis. Through its enzymatic activity, CD26 cleaves a plethora of proteins. In cancer, CD26 can serve as either a tumor suppressor or activator depending on the tumor microenvironment. CD26-expressing tumors are thus associated with better or worse patient survival. For example, CD26 expression in malignant pleural mesothelioma has been shown to be associated with prolonged survival (4), while in colorectal cancer, a higher expression of CD26 was unfavorably correlated with survival (5).

For almost 20 years, inhibitors of CD26 have been employed in routine clinical use for the treatment of type 2 diabetes. CD26 inhibitors exert their function by modulating GLP1 levels, thus enhancing the availability of insulin. Recent studies, among them a meta-analysis on more than 100 randomized trials, have surprisingly revealed that patients who take a CD26 inhibitor for the treatment of type 2 diabetes showed a decreased risk for the development of neoplasms (6). Other cohort studies on cancer patients with CD26 inhibitor intake have shown a better long-term outcome in colorectal (7) or prostate cancers (8), while patients with pancreatic or breast cancer did not have this favorable effect (8). The better prognosis in patients with colorectal cancer taking CD26 inhibitors was attributed to a change in their immune cell profile (7). Given the catalytic activity of CD26 on chemokines that guide effector T cells towards tumors, Ng and colleagues suggested that a decreased degradation of these chemokines enhances the trafficking of anti-tumor T cells into solid tumors, resulting in a better outcome (7). Bishnoi and colleagues also found in lung cancer patients that the combined use of dipeptidyl peptidase 4 (DPP4) inhibitors and metformin can significantly increase survival (9).

Within the tumor environment, CD26 can also exert a pro-tumorigenic activity through the induction of epithelial-to-mesenchymal transition (EMT) with an upregulation of EMT markers such as N-cadherin (10, 11). In one of our previous studies in mouse lung cancer models, we found that the treatment with a CD26 inhibitor suppresses lung metastases and primary lung cancer growth by attenuating EMT and enhancing tumor-infiltrating NK cells (12, 13). Others have shown that the downregulation of CD26 suppresses tumors in lung and mesothelioma models by modifying the EMT pathway, which is in line with our data (14, 15).

In this study, we investigated the expression of CD26 along with EMT markers in patients with non-small cell lung cancer (NSCLC) and correlated it with the clinical course. Moreover, we tested CD26 inhibition on EMT markers and lung cancer cells *in vitro*.

## Patients and methods

### Patient cohort

Surgically resected lung tumor specimens from pathologically confirmed primary, NSCLC patients were collected between 1999 and 2002 at the University Hospital Zurich (USZ). Clinical data were extracted from the medical records at the USZ. Tumor histopathological staging and histological subtyping were performed according to the seventh edition of the TNM staging system for lung cancer (16, 17). To confirm histological diagnosis, TTF-1 and p40 immunohistochemistry were performed on tissue microarrays (TMAs).

Histological tumor types of the USZ cohort were adenocarcinoma (n = 463/51.2%), squamous cell carcinoma (n = 388/42.9%), large-cell carcinoma (n = 24/2.6%), and adenosquamous carcinoma (n = 29/3.2%). All patients provided signed informed consent, and the Zurich Ethical Commission approved the study protocol (StV29-2009).

### Immunohistochemistry on tissue microarrays

Formalin-fixed paraffin-embedded tissue blocks were retrieved from the surgical pathology archives, and TMAs were constructed from NSCLC tumor tissue of the enrolled patients using a tissue arrayer. Representative tissue cores of 0.6 mm in diameter were taken in duplicates for each patient. Immunohistochemical stains were performed on 4-μm sections on a Ventana Benchmark Ultra using an OptiView DAB Kit (Ventana Chicago, Illinois, US). For Versican staining, a ChromoMap DAB Kit was used. CD26 staining was performed on a Bond/Leica platform using a Bond Polymer Refine Kit DS9800. Antibodies against the following proteins were used: CD26 (1:100 dilution; Cell Signaling, Danvers, MA, US; #67138), Periostin (1:10; Abcam, Cambridge, MA, US; #ab14041), Vimentin (1:250 dilution; Dako, Glostrup, Denmark; M7020), Versican (1:500 dilution; Seikagaku Corporation, Tokyo, Japan; #270428), E-cadherin (1:200 dilution; Cell Marque, Rocklin, CA, US; #246R-16), and β-catenin (1:50 dilution; BD Biosciences, San Jose, CA, US; #610154). Elastica van Gieson staining was performed according to the standard technique.

### Immunohistochemistry and TMA scoring and analyses

**Figure 1:**
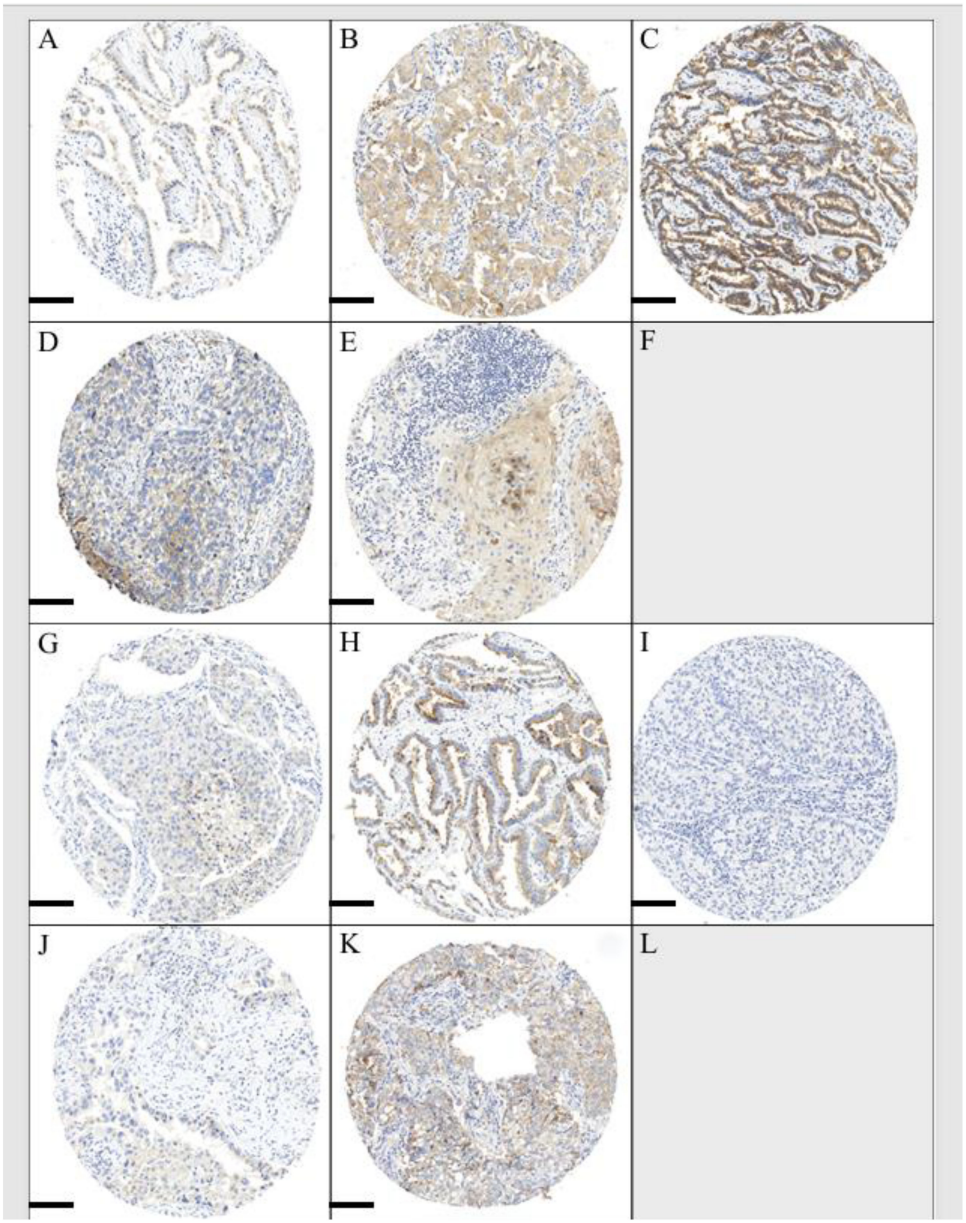
Scoring of CD26 immunohistochemistry. **(A–C)** Adenocarcinoma scores 1, 2, and 3. **(D–F)** Squamous cell carcinoma score 1, 2, and no score 3 (F; blank). **(G)** Adenosquamous score 1. **(H, I)** Same case of adenosquamous carcinoma with **(H)** adenocarcinoma component score 2 and **(I)** squamous cell component score 0. **(J–L)** Large-cell carcinoma score 1, 2, and no score 3 (blank). Scale bar, 100 µm.

CD26 expression and the EMT epithelial cell marker E-cadherin were scored on cancer cells showing membranous and cytoplasmic staining. The EMT markers Vimentin, β-catenin, Periostin, and Versican were scored within the stroma and intracellular compartment as previously reported (18). All markers were scored semi-quantitatively as 0 (negative), 1 (low), 2 (moderate), or 3 (high). CD26 immunohistochemical expression scoring in different NSCLC histological subtypes can be seen in **Figure 1** (A–C, adenocarcinoma; D–F, squamous cell carcinoma; G–I, adenosquamous; J–L, large-cell carcinoma).

Normal lung tissue that was derived from a distant site from the tumor served as a control for the comparison of staining between TMAs. Similarly, Elastica van Gieson staining was scored by collagen density (red) and elastic (black) fibers in the desmoplastic intratumoral stroma and was scored semi-quantitatively as 0 (no fibers), 1 (few), 2 (moderate), or 3 (high fiber amount), as previously reported (18). For each patient, the scores from both cores were added to obtain an overall score of 0 to 6, which was used for statistical analysis. The scoring was performed by an experienced pathologist (MH) in a blinded manner. Data from public data portals cBioPortal (https://www.cbioportal.org/) and DepMap Portal (https://depmap.org/portal) were used to corroborate the potential correlation of mRNA genes between EMT phase markers and DPP4.

### Tumor cell lines

The mouse cell line Lewis lung carcinoma (LLC) was purchased from American Type Culture Collection (Manassas, VA, USA). The human NCI-60 cell lines (H460, A549, H2347, HOP62, H1347, and H522) were obtained from Charles River (Boston, MA, USA) under a material transfer agreement with the National Cancer Institute (Bethesda, MD, USA). Upon arrival, cell lines were stored at early passages (<3) in liquid nitrogen and were used in the experiments for no more than 3 months. Mai9 and Gon8 were derived from malignant pleural effusions of NSCLC patients (19). All cell lines were cultivated in Dulbecco's Modified Eagle Medium (DMEM) containing 10% Fetal Bovine Serum (FBS) and penicillin/streptomycin within 5% CO_2_ chamber.

### Flow cytometry

LLC and H460 cell lines (1 × 10^5^ cells per well) were seeded on a 24-well plate for a 3D culture format. 3D cell cultures were chosen because 3D culture systems optimally provide conditions to best analyze the intercellular space, in which EMT markers are spatially located. A 1% agarose gel was coated on a 24-well plate to establish spheroid formation with LLC and H460 cell lines (**Supplementary Figures 3A, B**, respectively) in a 3D culture format. At day 3 after vildagliptin treatment, monolayer or 3D cultured spheroids were dissociated with Accutase and filtered through a 70-μm cell strainer. Fixable Viability Dye (Thermo Fisher Scientific, Waltham, MA, US) for dead cell stain and EMT-related protein antibodies against Vimentin and N-cadherin (Thermo Fisher Scientific, Waltham, MA, US) were used for the analysis of tested cells using the BD CANTO flow cytometer. The quantitation and separation gating, including singlet identification (**Supplementary Figures 3C, D**), were performed employing FlowJo.

### Colony formation assay

The tumor cell proliferation of the human lung cancer cell line H460 and the murine LLC cell line was analyzed using the colony formation assay (CFA). We showed previously that both cell lines displayed CD26 expression with tumorigenic properties *in vivo* (12). Cells were seeded at a density of 500 cells per 9.5 cm^2^ onto 6-well plates, and the CD26 inhibitor vildagliptin was administered at a dosage between 10 and 160 µM. After 10 days of seeding under vildagliptin treatment, the number of cell colonies was counted microscopically.

### MTT assay

Cell lines were seeded into 24-well plates to reach 70% confluence. Two days after cell seeding, the media were exchanged with serum-free medium. The viability of cell lines upon vildagliptin treatment was assessed using the methylthiazolyldiphenyl-tetrazolium bromide (MTT; Sigma-Aldrich, Burlington, MA, US) assay.

### Statistics

Histological scoring data are presented as dot plots. Data from the colony formation assay, MTT assay, and flow cytometry are presented as mean ± standard deviation. Statistical analysis was performed using GraphPad Prism 9 (GraphPad Software Inc., La Jolla, CA, USA) and the R software package. Student’s t-test, Kruskal–Wallis test with Dunn’s multiple comparisons, Mann–Whitney U-test, Spearman’s rank test, log-rank (Mantel–Cox) test, and Cox proportional hazards model were used for statistical analysis, as indicated. A p-value <0.05 was regarded as statistically significant.

## Results

### Patient characteristics

A total of 904 NSCLC samples and respective clinical data were analyzed. The study population included 463 patients (51.2%) with adenocarcinoma and 388 patients with squamous cell carcinoma (42.9%); Furthermore, 29 patients (3.2%) with adenosquamous carcinoma and 24 patients (2.7%) with large-cell carcinoma were included. Patient characteristics stratified by tumor subtype are summarized in the **Supplementary Table**.

### CD26 is preferentially expressed in lung adenocarcinoma

**Figure 2:**
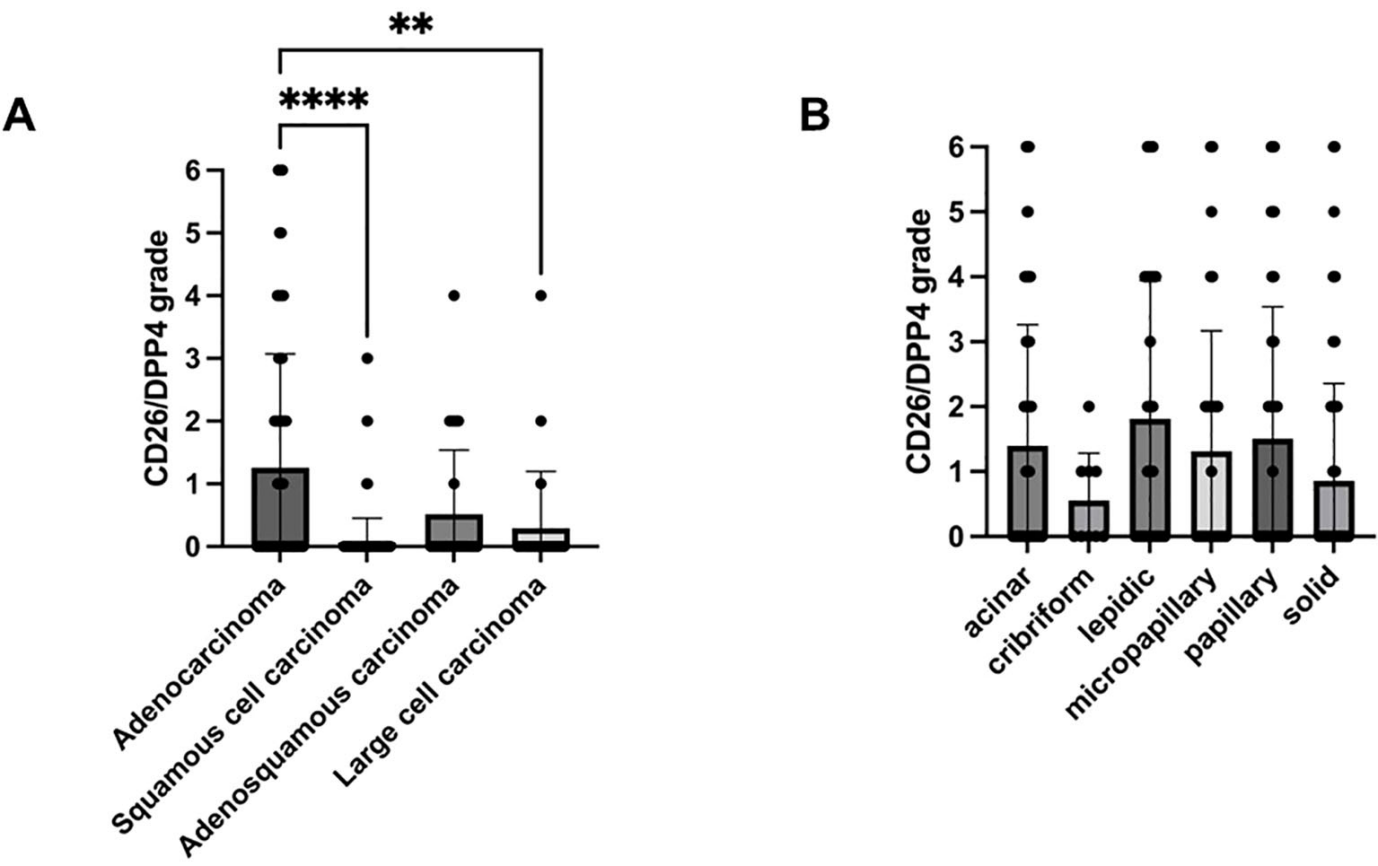
CD26 expression among NSCLC subtypes. Adenocarcinoma expresses significantly more CD26 compared to squamous cell carcinoma (p < 0.0001) and large-cell carcinoma (p = 0.0049). **(A)** Comparison between adenocarcinoma and adenosquamous carcinoma did not reach statistical significance (p = 0.1104). **(B)** Comparison of CD26 expression across adenocarcinoma subtypes. Although the overall Kruskal–Wallis test indicated statistical significance (p = 0.0493), none of the pairwise *post-hoc* comparisons reached significance after correction for multiple testing, suggesting only modest differences among subtypes without robust differences between individual groups. Kruskal–Wallis test, Dunn’s multiple comparisons. **p < 0.01; ****p < 0.0001. NSCLC, non-small cell lung cancer.

The CD26 immunohistochemical expression in different histological NSCLC subtypes was evaluated (**Figures 1**, **2**). Membranous and cytoplasmic expression intensity for CD26 was scored the highest in pulmonary adenocarcinoma as compared to other histological subtypes (**Figure 2A**). Specifically, CD26 expression was significantly higher in adenocarcinoma compared to squamous cell carcinoma (p < 0.0001). In particular, tissue cores from squamous cell carcinomas, adenosquamous or large-cell carcinomas did not show a CD26 expression of a score higher than 2 (**Figures 1F, G–I, L**). However, CD26 expression was only detected in 41.90% of adenocarcinoma samples, while 58.10% remained negative for CD26 expression.

To further dissect adenocarcinoma subtypes, we compared CD26 expression across different histological adenocarcinoma variants (**Figure 2B**). Although the overall Kruskal–Wallis test indicated statistical significance (p = 0.0493), none of the pairwise *post-hoc* comparisons reached significance after correction for multiple testing. This suggests that, while there is modest evidence for differences among adenocarcinoma subtypes as a whole, no robust differences could be confirmed between individual subgroups. We therefore concluded that CD26 expression is not directly associated with adenocarcinoma subtype.

Furthermore, we stratified adenocarcinoma patients by CD26 expression. Adenocarcinoma patient characteristics are summarized in **Table 1**. Importantly, the adenocarcinoma subgroups did not show significant differences regarding patient age, gender, Union internationale contre le cancer (UICC) tumor stage, histological grading, or vascular and pleural infiltration.

**Table 1:** Clinical and pathological parameters of adenocarcinoma groups and grade of CD26 expression.

			CD26/DPP4 negative	CD26/DPP4 positive	p
			269	194	
Age (years)	Mean (SD)		63.01 (10.50)	64.01 (10.14)	0.308
Gender	n (%)	Male	163 (61.0)	107 (55.4)	0.267
		Female	104 (39.0)	86 (44.6)	
Grade	n (%)	G1	20 (7.5)	24 (12.4)	0.177
		G2	119 (44.6)	86 (44.6)	
		G3	128 (47.9)	83 (43.0)	
Vascular invasion	n (%)	Negative	173 (64.8)	119 (61.7)	0.554
		Positive	94 (35.2)	74 (38.3)	
Pleural invasion	n (%)	Negative	152 (56.9)	126 (65.3)	0.087
		Positive	115 (43.1)	67 (34.7)	
UICC Stage	n (%)	IA	41 (15.4)	42 (21.8)	0.217
		IB	51 (19.1)	37 (19.2)	
		IIA	35 (13.1)	33 (17.1)	
		IIB	30 (11.2)	12 (6.2)	
		IIIA	81 (30.3)	50 (25.9)	
		IIIB	9 (3.4)	4 (2.1)	
		IV	20 (7.5)	15 (7.8)	

### CD26 expression is an independent prognostic factor for survival in lung adenocarcinoma

**Figure 3:**
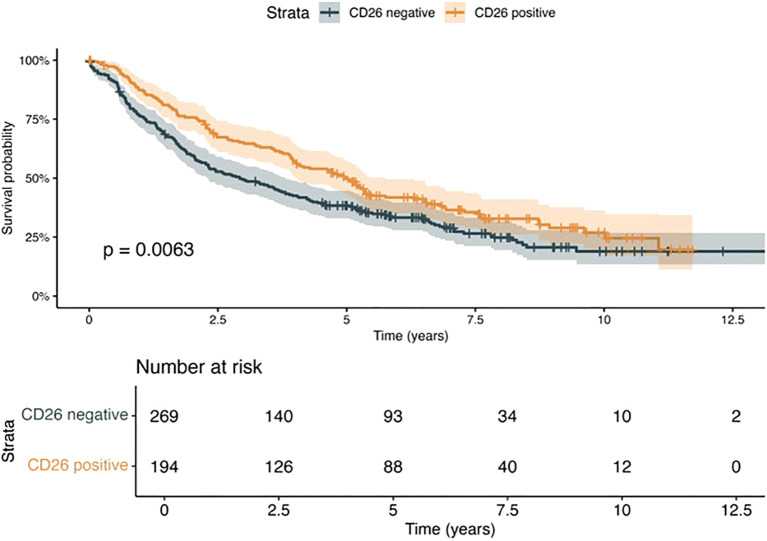
Survival function of adenocarcinoma patients estimated by Kaplan–Meier method, stratified by immunohistochemical confirmation of CD26/DPP4 expression (CD26-positive vs. CD26-negative). Time is displayed in months. Patients with CD26/DPP4-expressing tumors show a survival benefit compared to their CD26/DPP4-negative counterparts (n = 463, p = 0.0063, log-rank test).

Analyzing the two adenocarcinoma subgroups, we found that the overall survival of adenocarcinoma patients was significantly impacted by CD26 expression (**Figure 3**). The mean survival time of patients suffering from CD26-positive tumors and those with CD26-negative tumors was 6.10 and 4.95 years, respectively (p = 0.0063). Furthermore, Cox proportional hazards regression revealed positive CD26 expression as a protective factor for survival, independent from patient age, tumor grading, tumor stage, and vascular or pleural infiltration (p = 0.0022; **Table 2**).

**Table 2:** Cox proportional hazards model reveals CD26 positivity on immunohistochemistry as an independent protective factor for survival.

Covariate	Coefficient	Hazard ratio	Confidence interval	p	
Age	0.0124	1.0125	1.0014–1.0237	0.0274	*
G2 grade	0.2439	1.2762	0.8102–2.0103	0.2928	
G3 grade	0.3075	1.3600	0.8632–2.1425	0.1849	
Positive vascular invasion	0.3775	1.4587	1.1406–1.8654	0.0026	**
Positive pleural invasion	−0.0513	0.9500	0.7450–1.2114	0.6789	
UICC Stage IB	−0.0409	0.9599	0.6236–1.4776	0.8524	
UICC Stage IIA	0.4210	1.5235	0.9831–2.3609	0.0596	
UICC Stage IIB	0.4887	1.6302	0.9841–2.7003	0.0577	
UICC Stage IIIA	0.8966	2.4512	1.6851–3.5655	2.74 × 10^−6^	***
UICC Stage IIIB	1.2985	3.6639	1.8667–7.1916	0.0002	***
UICC Stage IV	1.9028	6.7044	4.0609–11.0689	1.02 × 10^−13^	***
CD26/DPP4 positivity	−0.3628	0.6958	0.5515–0.8778	0.0022	**

*p < 0.05; **p < 0.01; ***p < 0.001.

### The expression of CD26 correlates with EMT marker expression in adenocarcinoma

**Figure 4:**
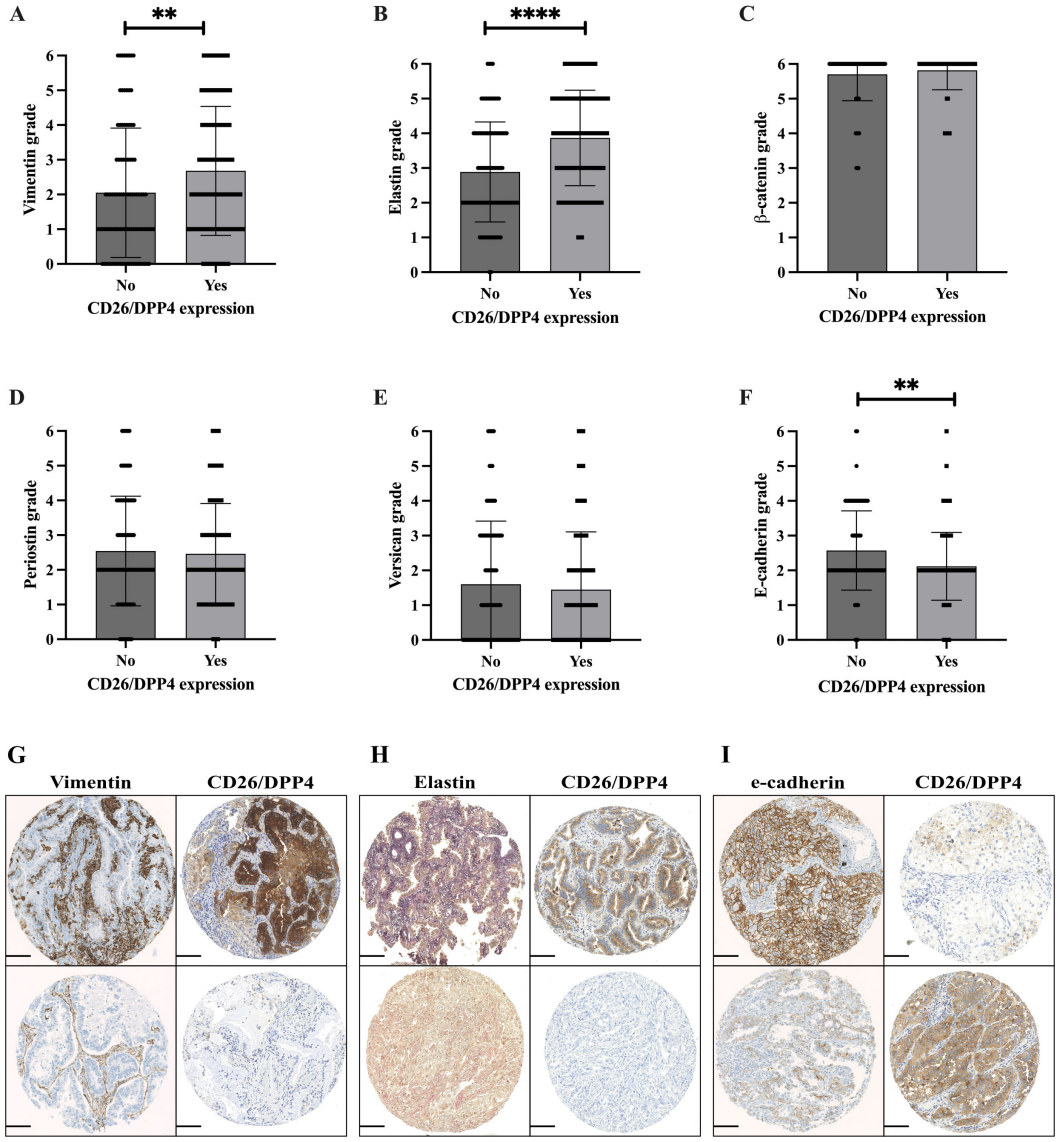
EMT marker analysis in adenocarcinoma in correlation with CD26/DPP4 expression, Vimentin, Elastin, β-catenin, Periostin, Versican, and E-cadherin **(A–F)**. Mann–Whitney U-test; **p < 0.01; ****p < 0.0001. **(G–I)** Serial section analysis of representative cases; each row represents a single case with various staining scores for specific markers. **(G)** Upper row: Vimentin score 3/CD26/DPP4 score 3. Lower row: Vimentin score 1/CD26/DPP4 negative. **(H)** Upper row: Elastin score 3, CD26/DPP4 score 2. Lower row: Elastin score 1, CD26/DPP4 negative. **(I)** Upper row: E-cadherin score 3, CD26/DPP4 score 1. Lower row: E-cadherin score 1, CD26/DPP4 score 3. Scale bar, 100 µm. EMT, epithelial-to-mesenchymal transition.

As CD26 expression can influence EMT induction in cancer, we analyzed the correlation of CD26 with the EMT phase markers E-cadherin, Vimentin, β-catenin, Elastin, Periostin, and Versican. CD26 expression correlated with the expression of the mesenchymal stem cell markers Vimentin, β-catenin, and Elastin, while the epithelial marker E-cadherin was inversely correlated (**Table 3**). The comparison of EMT marker expression between the CD26-positive and CD26-negative adenocarcinoma subgroups is shown in **Figures 4A–I**. The levels of Vimentin and Elastin were significantly higher in CD26-positive tumors compared to CD26-negative adenocarcinoma tissues (**Figures 4A, B**; p = 0.0027 and p < 0.0001, respectively). Conversely, E-cadherin was significantly lower in CD26-positive tumors (**Figure 4F**; p = 0.0021). The analysis of the serial sections of individual cases demonstrated identical results for Vimentin, Elastin, and E-cadherin (**Figures 4G–I**). For example, **Figure 4G** depicts two cases. In the upper row, high expression of Vimentin (score of 3) and CD26 (score of 3) are found in the same case. In contrast, the case in the lower row exhibits a Vimentin score of only 1 and is CD26-negative.

**Table 3:** Correlation between expression of CD26 and EMT-related proteins.

EMT-related proteins	Spearman's r	p	
Elastin	0.3425	<0.0001	****
Vimentin	0.1730	0.0081	**
β-Catenin	0.1421	0.0301	*
Periostin	−0.13	0.41	
Versican	−0.09719	0.1391	
E-Cadherin	−0.2149	0.0010	*** §

EMT, epithelial-to-mesenchymal transition.

Spearman's rank test. *p < 0.05; **p < 0.01; ***p < 0.001; ****p < 0.0001; § inversely correlated.

Public data cBioPortal (https://www.cbioportal.org/) and DepMap Portal (https://depmap.org/portal) reveal a correlation of the mRNA gene expression of the EMT phase markers E-cadherin, Vimentin, β-catenin, Elastin, Periostin, and Versican with DPP4 in lung adenocarcinoma (**Supplementary Figures 1A–F**, **Supplementary Figures 2A–F**), thus corroborating our immunohistochemical data.

### Treatment with the CD26 inhibitor vildagliptin reduces the self-renewal capacity of cancer cells *in vitro*

**Figure 5:**
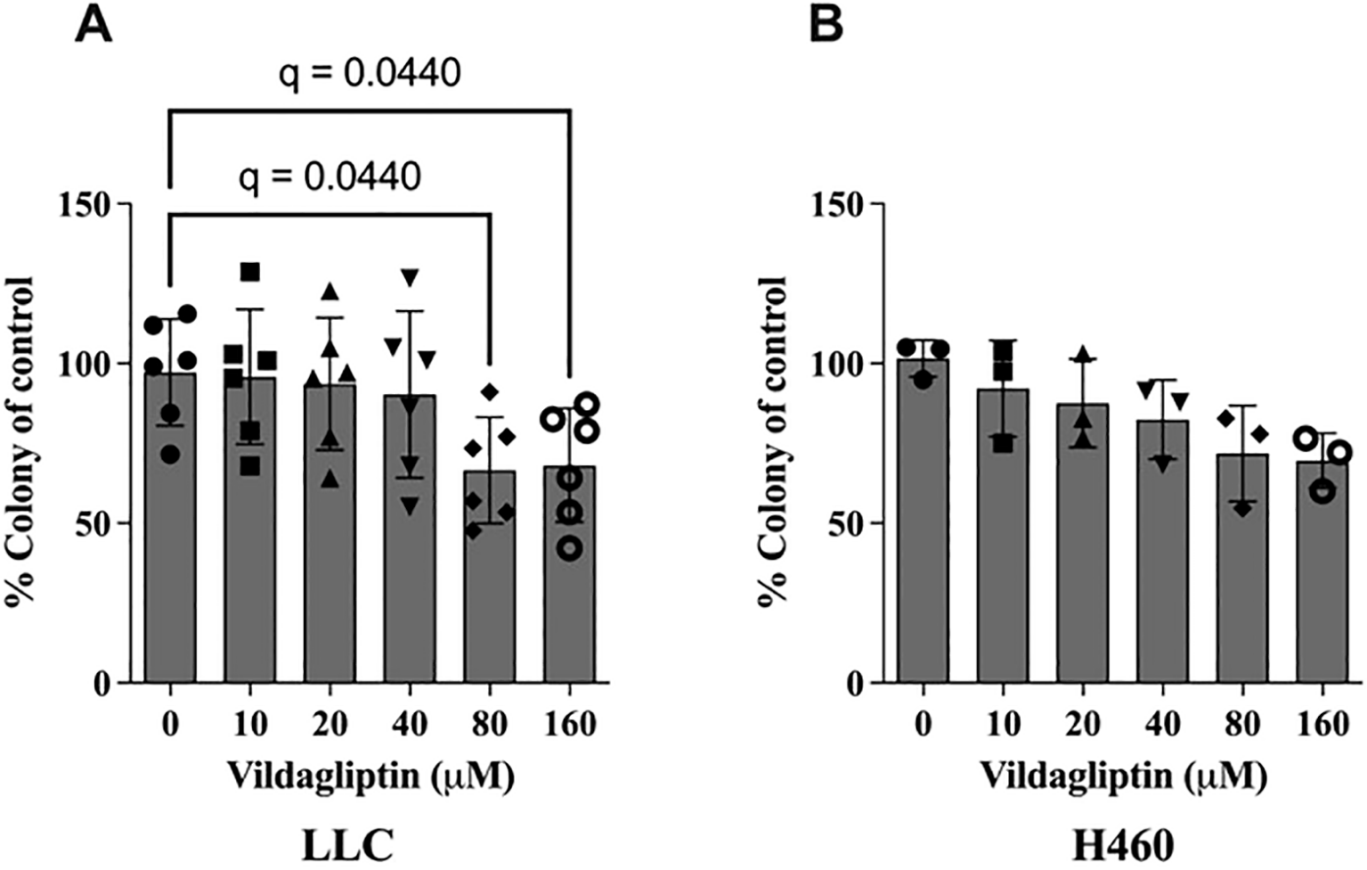
CD26 inhibition by vildagliptin showed reduced colony formation in a dose-dependent manner in the CD26-expressing mouse lung cancer cell line LLC. **(A)** Mean ± standard deviation, one-way ANOVA, p = 0.0279, False Discovery Rate (FDR) method by Benjamini–Hochberg. In the CD26-positive human lung cancer cell line H460, we observed a similar trend. **(B)** Mean ± standard deviation, one-way ANOVA, p = 0.0579. LLC, Lewis lung carcinoma.

Colony formation is an EMT-associated cancer stem cell feature (20, 21). Thus, we tested whether treatment with the CD26 inhibitor vildagliptin could modulate colony formation *in vitro*. Here, we found that vildagliptin treatment reduced the proliferation and viability of both lung cancer cell lines, LLC and H460 (**Figures 5A, B**). However, additional analyses in other human NSCLC cell lines showed only an effect in the Mai9 cell line, but not in the A549 or Gon8 cell line (**Supplementary Figure 4A**). Also, vildagliptin showed no direct cytotoxic effect at 10 and 100 µM in other human NSCLC cell lines (**Supplementary Figure 4B**).

### EMT regulation by pharmacological CD26 inhibition

**Figure 6:**
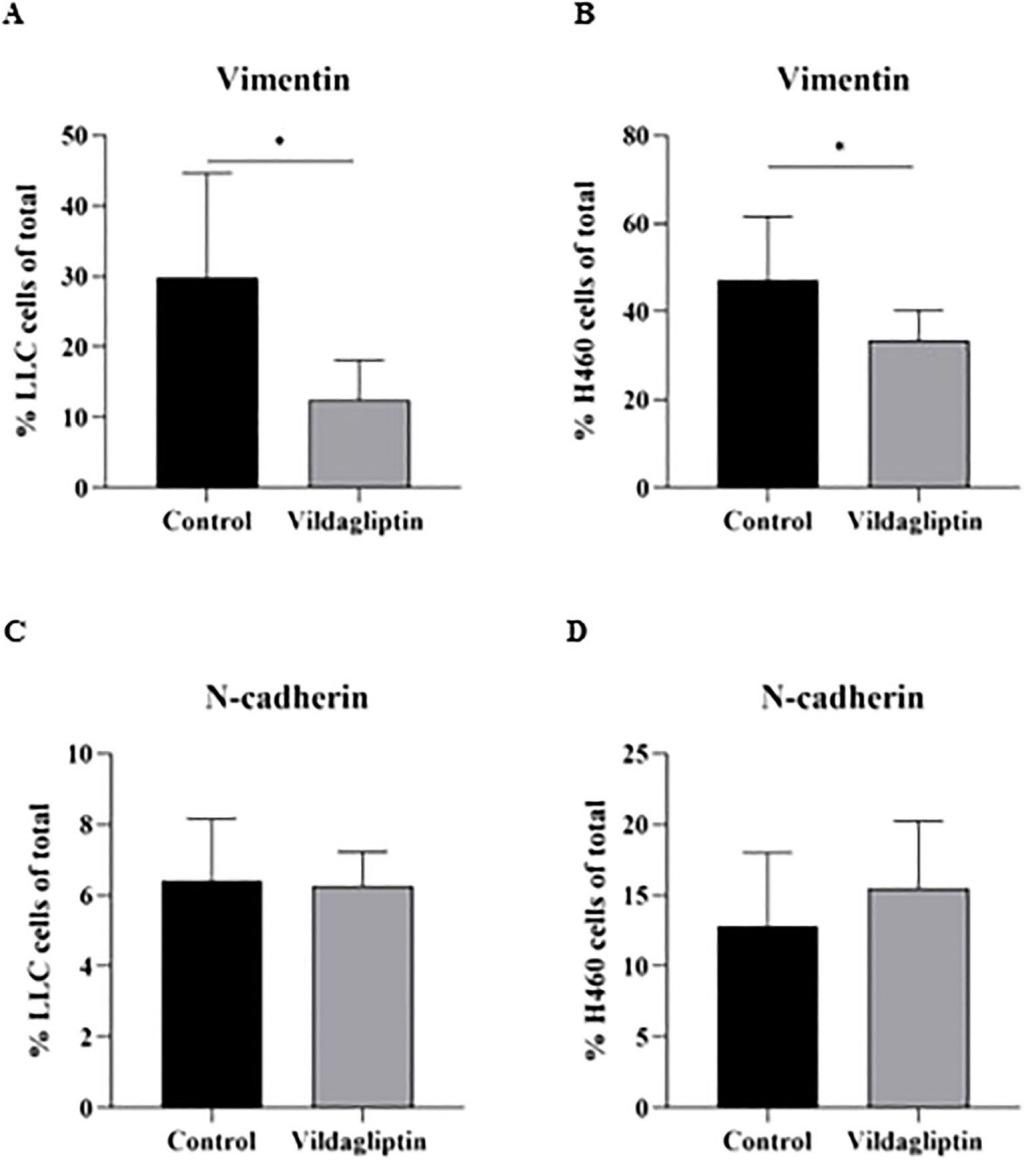
Pharmacological CD26 inhibition reduced the expression of the EMT marker Vimentin in the lung cancer cell lines LLC and H460, tested in 3D culture conditions **(A, B)**. N-cadherin remained unchanged by vildagliptin treatment **(C, D)**. Student’s t-test: *p < 0.05. EMT, epithelial-to-mesenchymal transition; LLC, Lewis lung carcinoma.

Vildagliptin treatment reduced the expression of Vimentin in both LLC and H460 cell lines in a 3D spheroid culture format (**Figures 6A, B**, respectively, and **Supplementary Figure 3E**); however, N-cadherin did not change upon CD26 inhibition (**Figures 6C, D**).

## Discussion

CD26 has gained increased interest in cancer research due to its relevant role in immunomodulation and tumorigenesis (22). In this study, we analyzed the expression of CD26 in lung cancer in a large cohort of patients and demonstrated that CD26 is significantly overexpressed in lung adenocarcinoma and correlates with better overall patient survival. Furthermore, CD26-expressing adenocarcinomas showed a higher expression of the EMT phase markers Vimentin and Elastin. *In vitro*, the pharmacological inhibition of CD26 reduced Vimentin expression and had an anti-proliferative effect in tumor cell lines.

Various malignancies have been shown to express CD26, e.g., hepatocellular carcinoma, thyroid carcinoma, renal cell carcinoma, prostate cancer, gastrointestinal tumors, malignant mesothelioma, and some hematologic malignancies (22). CD26 expression on these tumors can have either a tumor-promoting or tumor-suppressing effect, depending on the individual tumor microenvironment, organ-specific signaling pathways, and mediator changes, e.g., hormones. It has been shown that a high expression of CD26 in colorectal cancer correlated with the presence of metastases, an advanced disease stage, and worse patient survival (5). For prostate cancer, dichotomous effects were found showing a higher expression of CD26 on cancer cells, which correlated with a poor prognosis of patients (23). However, *in vitro* analyses on prostate cancer showed that CD26 has a suppressor function on tumor progression by blocking the basic fibroblast growth factor signaling pathway (24) or suppressing the chemotactic migration of tumor cells (25).

In an elaborated histomorphological analysis on lung tumors, Asada and colleagues described CD26 to be expressed in higher levels in lung adenocarcinoma compared to other tumor types, such as squamous cell lung carcinoma (26). This study was the first of its kind, which is in line with the data we obtained in our patient cohort. In contrast, Wesley et al. found that the expression of CD26 is lost in different types of non-small cell lung carcinoma cell lines (27), thus assigning CD26 a tumor suppressor function in lung cancer. These dichotomous results could be owed to different culture conditions: Wesely and colleagues used tumor cell lines but not primary human cells. Also, their analyses were based on RNA and protein expression and did not employ histological samples.

In order to further dissect our immunohistochemical results, we analyzed the co-expression of CD26 with the markers of the epithelial-to-mesenchymal transition, a pathway that has been shown to promote tumor initiation, progression, and resistance to therapies (28). In fact, we found the phenomenon of partial EMT as an additional mechanism of tumor progression, development of metastases, and drug resistance (29) in which cells of the cancer and its environment co-express epithelial and mesenchymal markers. The expression of Vimentin and Elastin was positively correlated, while E-cadherin was negatively correlated with CD26, supporting the presence of partial EMT. Data from public data portals such as cBioPortal (https://www.cbioportal.org/) (30–32) and DepMap Portal (https://depmap.org/portal) (33) show the relation of mRNA gene expression between the EMT phase markers E-cadherin, Vimentin, β-catenin, Elastin, Periostin, and Versican to DPP4 in lung adenocarcinoma (**Supplementary Figures 1A–F**, **2A–F**). In part, EMT markers correlate with DPP4 and thus corroborate our immunohistochemical data.

The correlation between the overexpression of CD26 in adenocarcinoma histology and better patient survival that we found in our study is somewhat surprising and does not meet the general expectation that overexpressed molecules on tumor cells are targets for respective inhibitors or antibodies. Mechanisms such as those described in prostate cancer, for example, the inhibition of cell proliferation, increased p21 expression with subsequent apoptosis and cell cycle arrest, may assign CD26 to be a suppressor in tumors (24). However, increased CD26 expression within tumors harbors another, potentially favorable opportunity for an immune-modulating treatment: an increased density of CD26 within the tumor consequently leads to the increased enzymatic cleavage of tumor-relevant chemokines. The inhibition of CD26 then results in an enhancement of the local accumulation of these chemokines, thereby attracting anti-tumor cytotoxic T cells. This mechanism seems plausible, as it has already been shown by various groups (34–37). An increased expression of CD26 on mesothelioma has led to promising first phase I and II trials employing the CD26 antibody YS110 on pretreated patients and showed modest response but a good tolerance to the treatment (38, 39). Whether this is a direct effect on tumor cells or an indirect effect through the reduced cleavage of chemokines and enhanced T-cell attack towards the tumor is not clear from this study. Most recently, a combination therapy of metformin and DPP4 inhibitors with an immune checkpoint inhibitor (ICI) showed a significant improvement in clinical outcome without increasing the incidence of immune-related adverse events (irAEs) in NSCLC patients, thus supporting the concept of directing T cells against the tumor by two different, but synergistically acting, mechanisms (40).

For more than 15 years, CD26 inhibitors have been in routine use for the treatment of type 2 diabetes. There are a large number of retrospective studies on patient cohorts that were treated with a CD26 inhibitor for diabetes that analyze the incidence and course of different malignant diseases. Results obtained from these studies revealed distinct patterns. A recent multicentric analysis showed that the exposure to CD26 inhibitors led to a significantly higher progression-free survival in patients with advanced colorectal and airway cancers and a trend towards improved overall survival (41). Another recently released study on patients with type 2 diabetes who developed either a colorectal (n = 11,657) or lung cancer (n = 15,201), of which 1,876 patients took a gliptin, revealed improved overall survival in both cancer entities, with or without taking metformin (9). Other retrospectively conducted studies on patients with colorectal cancer show similar favorable results, indicating that gliptins induce specific immune responses leading to better survival (7). The first data on CD26-expressing primary lung cancer cells treated with the CD26 inhibitor vildagliptin that we present in this study showed that upon increasing the doses of vildagliptin, tumor cell colonies were reduced, indicating also here a tumor-inhibitory effect by a gliptin. Similar to human adenocarcinoma, the cell lines we used here also harbor the KRAS mutation, a mutation that can potentially impact the expression or function of CD26. Indeed, Grunt and colleagues found co-expression patterns of the stem cell markers CD133 and CD26 on KRAS-mutated HC116 colon cancer cells, showing that the co-expression of these markers enhances long-term growth and increased resistance to anticancer agents (42). However, there is no evidence from solid human tumors including lung cancer that supports this hypothesis.

So far, existing data about the expression and impact of CD26 in solid tumors are inconsistently reported; however, our data—and we consider this as a strength of this study—add more evidence to current literature on the role of CD26 and patient outcome in cancer in general and, for the first time, in lung adenocarcinoma in particular. Yet, our data are not uniform. On the one hand, we observed better survival in patients who express CD26; on the other hand, we observed a tumor-attenuating effect upon CD26 inhibition *in vitro*. Without doubt, both conditions harbor biases that could limit the significance of the one or the other result: CD26 was stained in tumor cells but not in infiltrating immune cells. A characterization of CD26 on immune cells could potentially give a hint of why patients have better survival. Also, the *in vitro* assays could potentially harbor biases: 2D and 3D spheroids were used, and even if aim-specified, results gained on these two different culture conditions should be interpreted with caution. Furthermore, the process of dissociation during Fluorescence Activated Cell Sorting (FACS) analysis induces an alteration of surface marker expression of molecules, thus again potentially affecting the results.

Nevertheless, with all its heterogeneity, this study can serve as a starting point to further dissect the role of CD26 as either a tumor-promoting or tumor-suppressing receptor.

## Data Availability

The raw data supporting the conclusions of this article will be made available by the authors, without undue reservation.

## Supplementary material

Supplementary Figure 1 Correlation of mRNA gene expression of the EMT phase markers E-cadherin, Vimentin, β-catenin, Elastin, Periostin, and Versican with DPP4 in lung adenocarcinoma **(A-F)**, retrieved from cBioPortal for Cancer Genomics (https://www.cbioportal.org/).

Supplementary Figure 2 Correlation of mRNA gene expression of the EMT phase markers E-cadherin, Vimentin, β-catenin, Elastin, Periostin, and Versican with DPP4 in lung adenocarcinoma **(A-F)** retrieved from Depmap Portal (https://depmap.org/portal).

Supplementary Figure 3 Analysis of the expression of EMT markers by Flow cytometry. Representative pictures of 3D spheroid formation of cell lines of LLC **(A)** and H460 **(B)** after 3 days of seeding. Dead cell and doublet exclusion gating **(C, D)**. Representative picture of anti-Vimentin antibody-stained populations of control (red), Vildagliptin treated (green), and unstained control (blue) with gating of Vimentin-positive cells (rectangular) **(E)**.

Supplementary Figure 4 CD26 inhibitor test on human NSCLC cell lines. Colony forming assay showed reduced stemness only on Mai9 cell line with Vildagliptin treatment (160µM) **(A)**. Vildagliptin showed no cytotoxic effect on human NSCLC cell lines with 10 and 100µM **(B)**. Student´s t-test: *p<0.05.
