# Supplementary material for: CD26 as a potential therapeutic target for lung adenocarcinoma
Source: Front Oncol. 2025 Dec 4;15:1552587. doi: 10.3389/fonc.2025.1552587 (PMC12711487; doi:10.3389/fonc.2025.1552587)
Supplement: Supplementary Figure 1 — Correlation of mRNA gene expression of the EMT phase markers E-cadherin, Vimentin, β-catenin, Elastin, Periostin, and Versican with DPP4 in lung adenocarcinoma (A-F), retrieved from cBioPortal for Cancer Genomics (https://www.cbioportal.org/). [file Table1.docx]

|  | **Adenocarcinoma** n=463 | **Squamous cell carcinoma**  n=388 | **Adenosquamous carcinoma**  n=29 | **Large cell carcinoma**  n=24 | **p** |
| --- | --- | --- | --- | --- | --- |
| **Age**  mean±SD | 63.43±10.36 | 65.19±9.30 | 64.28±9.68 | 63.29±10.8 | 0.076 |
| **Gender**  n (%) |  |  |  |  | <0.001 |
| **male** | 270 (58.7) | 309 (79.6) | 23 (79.3) | 13 (54.2) |  |
| **female** | 190 (41.3) | 79 (20.4) | 6 (20.7) | 11 (45.8) |  |
| **Histological Grading**  n (%) |  |  |  |  | <0.001 |
| **G1** | 44 (9.6) | 4 (1.0) | 0 (0.0) | 0 (0.0) |  |
| **G2** | 205 (44.6) | 184 (47.4) | 13 (44.8) | 0 (0.0) |  |
| **G3** | 211 (45.9) | 200 (51.5) | 16 (55.2) | 24 (100.0) |  |
| **Vessel invasion positive**  n (%) | 168 (63.5) | 145 (37.4) | 19 (65.5) | 3 (12.5) | 0.001 |
| **Pleural invasion positive**  n (%) | 182 (39.6) | 120 (30.9) | 14 (48.3) | 8 (33.3) | 0.031 |
| **UICC Stage**  n (%) |  |  |  |  | 0.026 |
| **IA** | 83 (18.0) | 54 (13.9) | 3 (10.3) | 0 (0.0) |  |
| **IB** | 88 (19.1) | 67 (17.3) | 5 (17.2) | 6 (25.0) |  |
| **IIA** | 68 (14.8) | 91 (23.5) | 3 (10.3) | 7 (29.2) |  |
| **IIB** | 42 (9.1) | 48 (12.4) | 3 (10.3) | 3 (12.5) |  |
| **IIIA** | 131 (28.5) | 100 (25.8) | 9 (31.0) | 5 (20.8) |  |
| **IIIB** | 13 (2.8) | 13 (3.4) | 1 (3.4) | 1 (4.2) |  |
| **IV** | 35 (7.6) | 15 (3.9) | 5 (17.2) | 0 (0.0) |  |

**Supplementary Table**. Clinical characteristics of all enrolled NSCLC patients stratified by tumour histology. Pearson’s Chi-squared test.
