# Supplementary material for: CD26 as a potential therapeutic target for lung adenocarcinoma
Source: Front Oncol. 2025 Dec 4;15:1552587. doi: 10.3389/fonc.2025.1552587 (PMC12711487; doi:10.3389/fonc.2025.1552587)
Supplement: Supplementary file 2 [file Presentation1.pptx]

## Slide 1
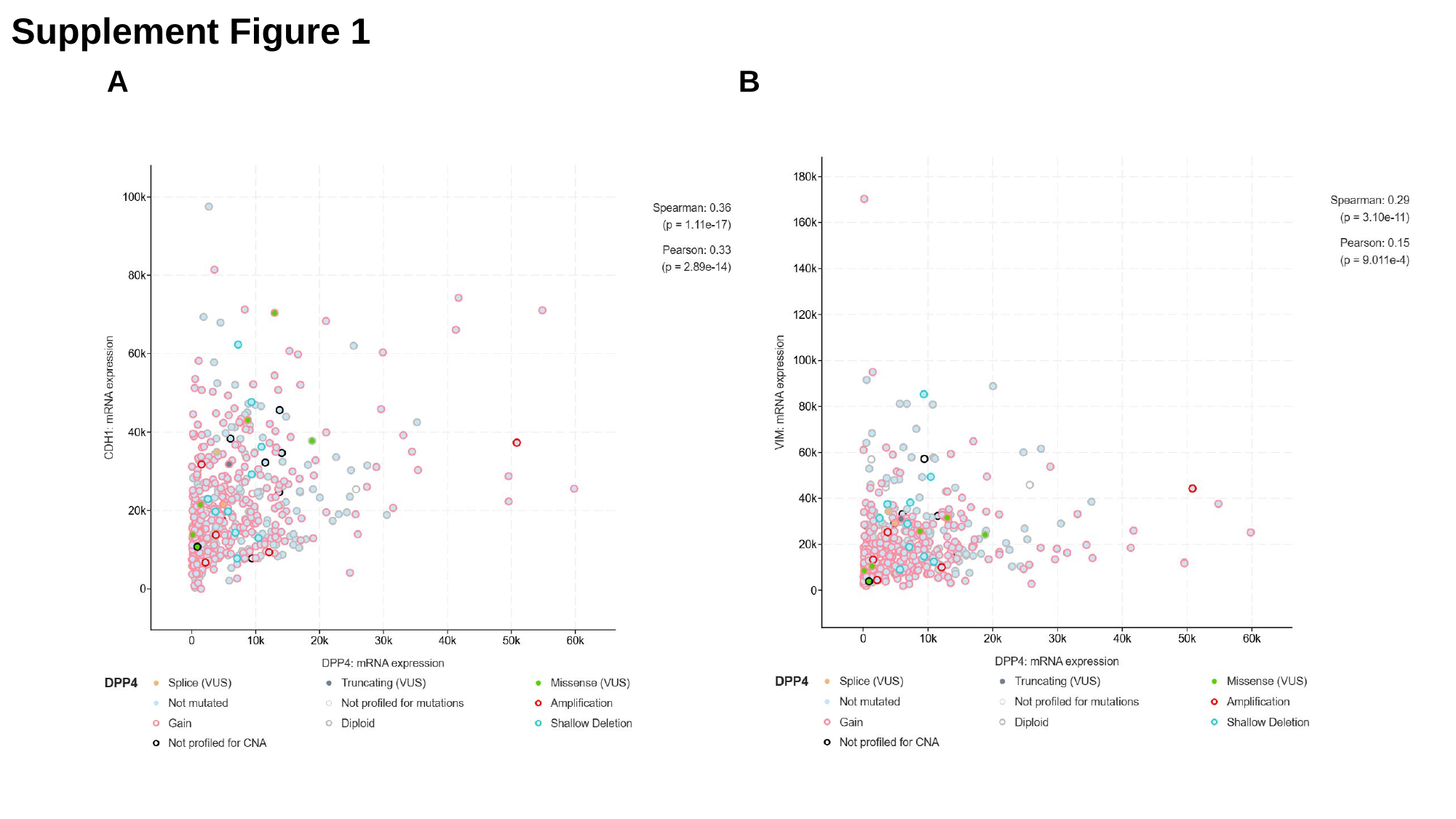

Supplement Figure 1
A
B

## Slide 2
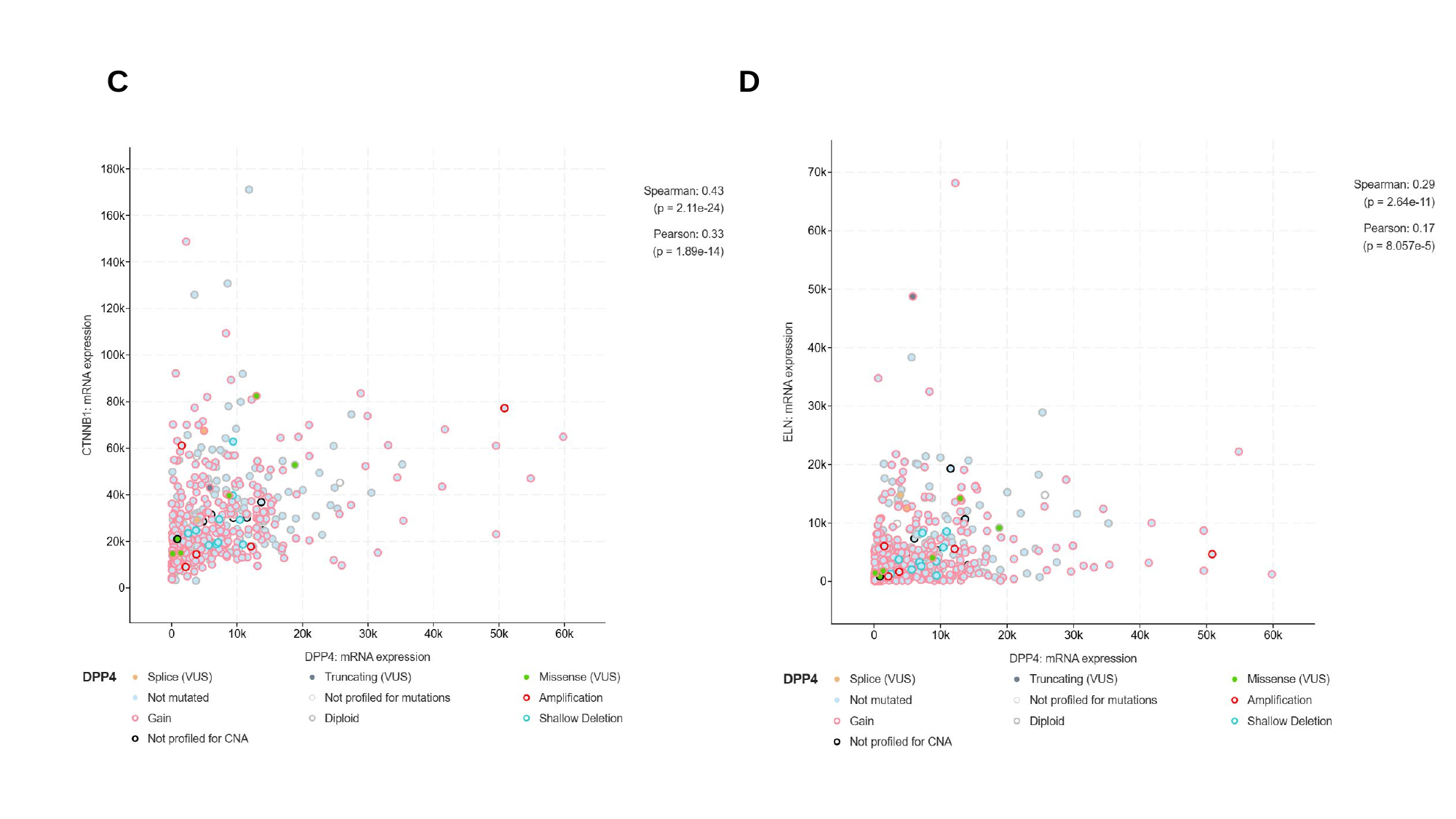

C
D

## Slide 3
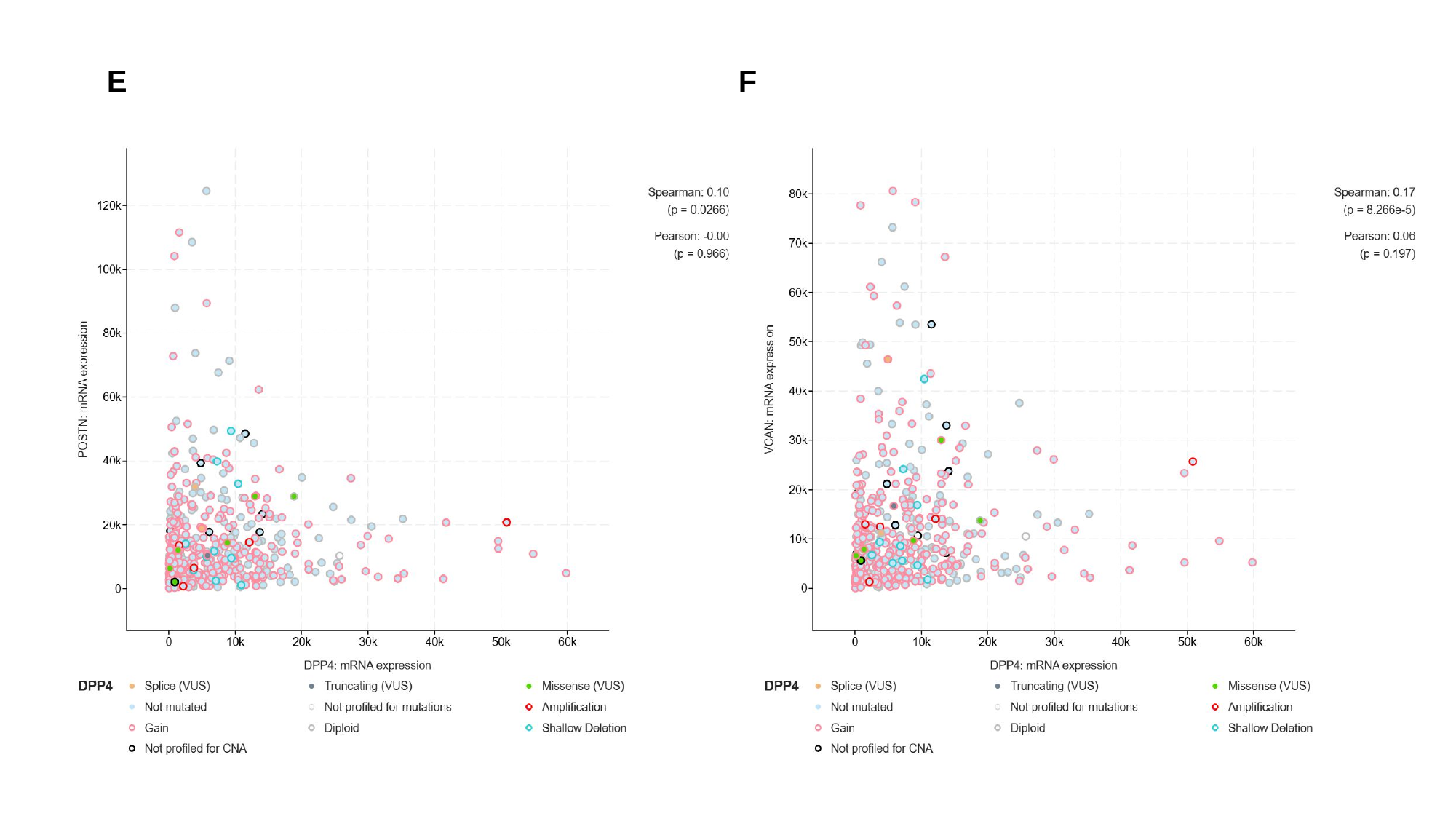

E
F

## Slide 4
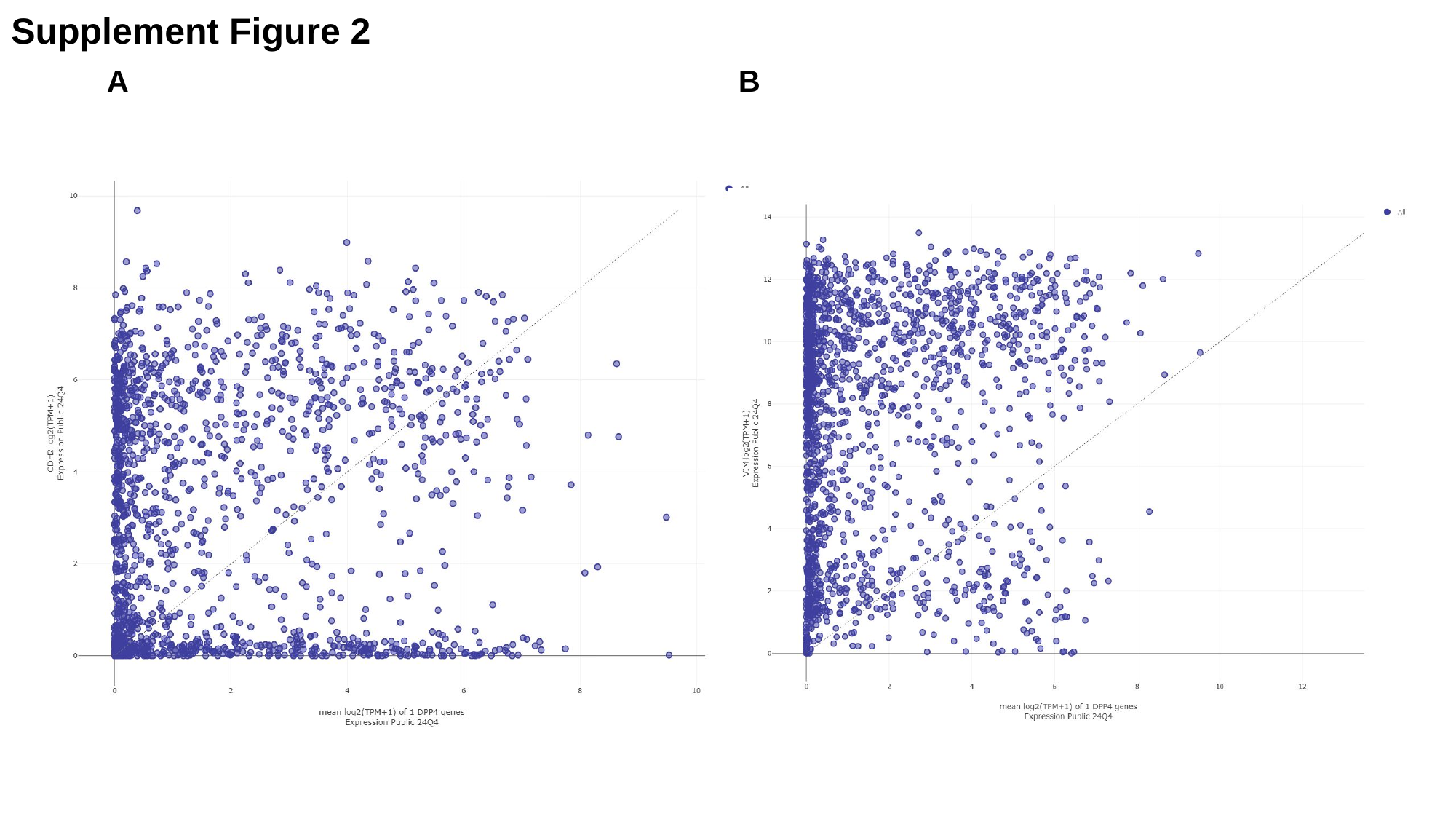

Supplement Figure 2
A
B

## Slide 5
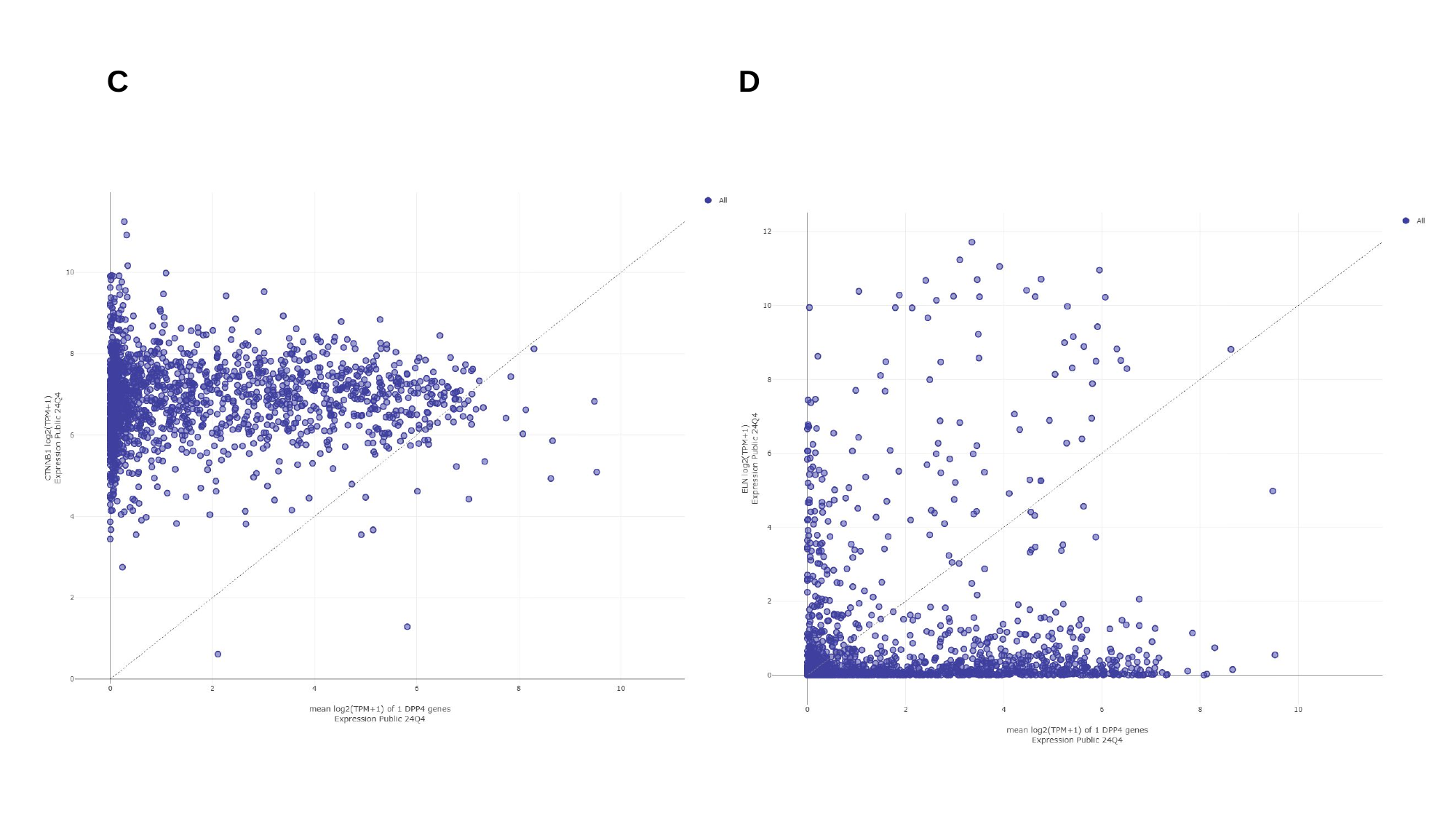

C
D

## Slide 6
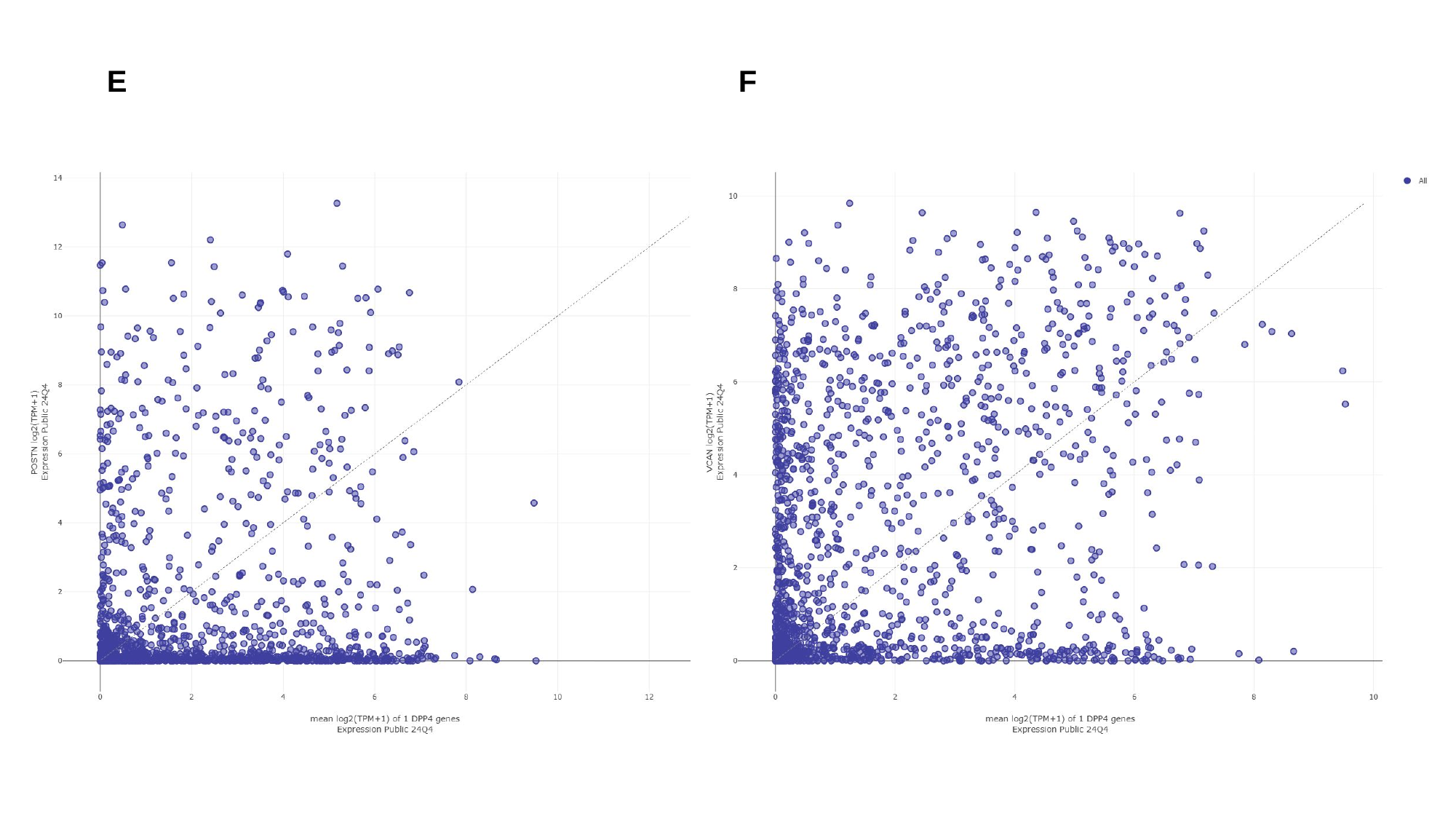

E
F

## Slide 7
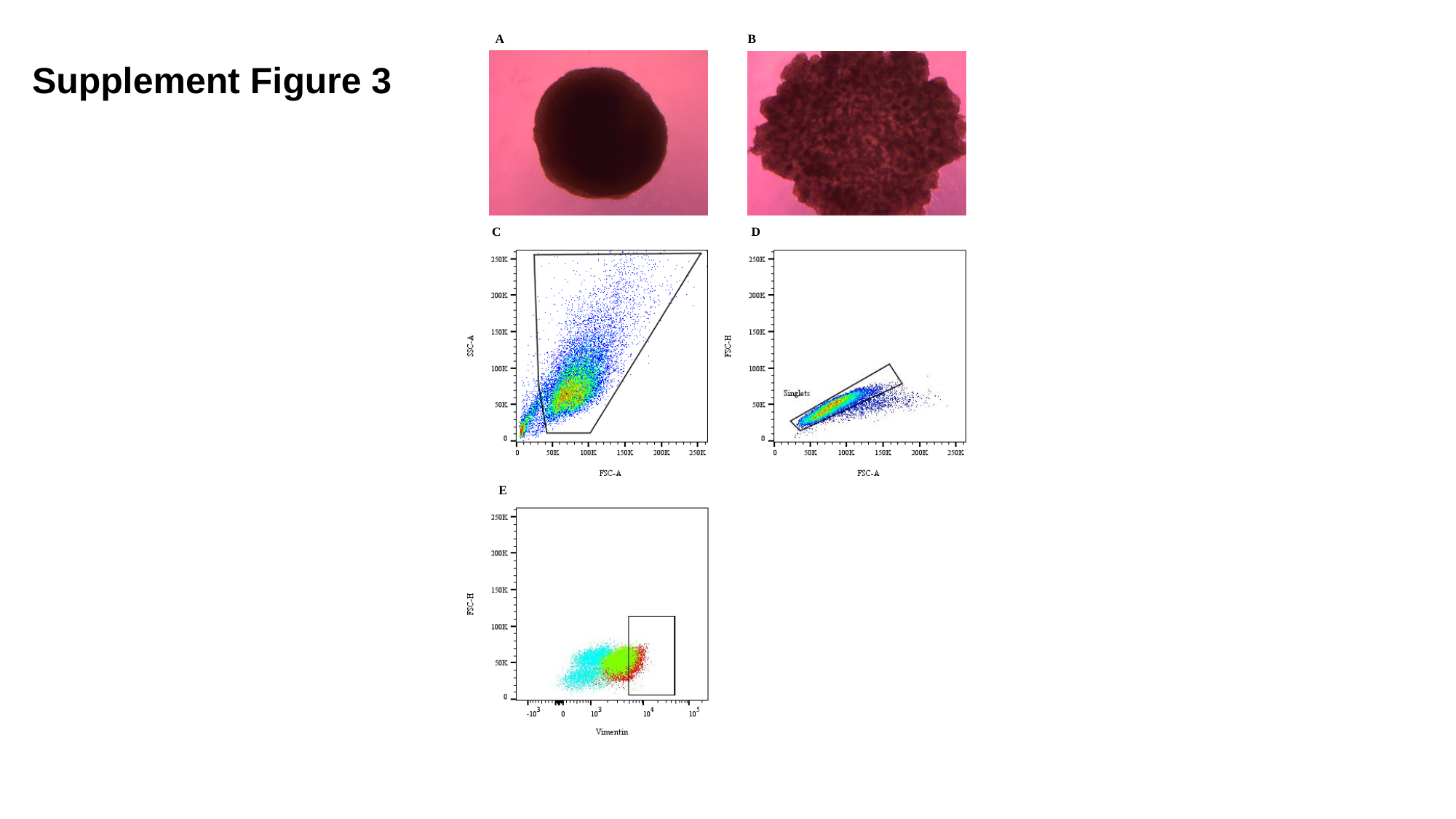

A B
Supplement Figure 3
 C D
 E

## Slide 8
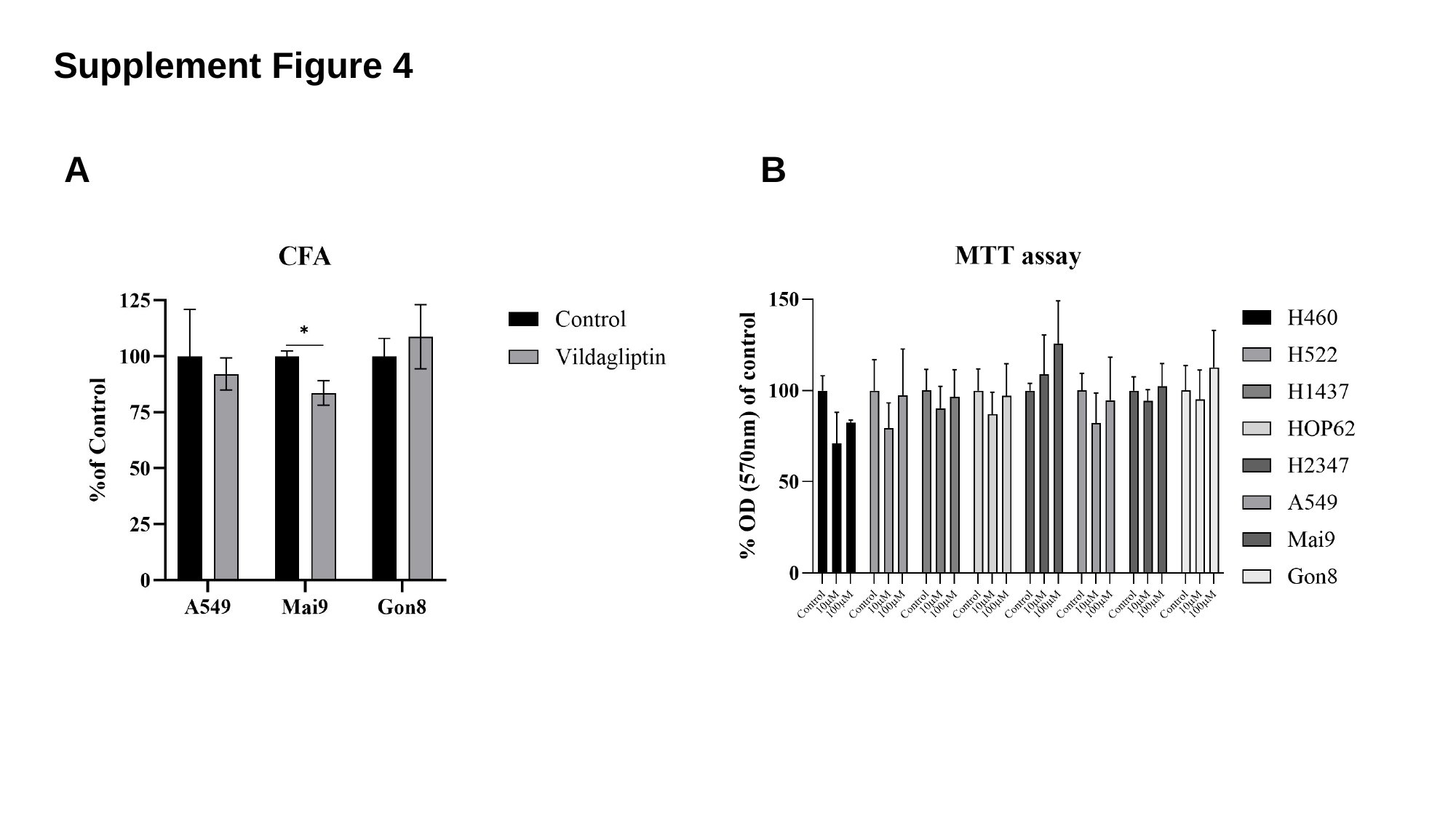

Supplement Figure 4
B
A
